# Supplementary material for: Modeling glioblastoma heterogeneity as a dynamic network of cell states
Source: Mol Syst Biol. 2021 Sep 16;17(9):e10105. doi: 10.15252/msb.202010105 (PMC8444284; doi:10.15252/msb.202010105)
Supplement: Supplementary file 5 — Source Data for Figure 3 [file MSB-17-e10105-s001.zip › Figure3A_sourcedata/GSEA_3065/nosigdb_state2.GseaPreranked.1622718419215/DOROSZKO_PERIVASCULAR_INVASION.html]

Details for gene set DOROSZKO\_PERIVASCULAR\_INVASION[GSEA]

|  || Dataset | state2 |
| Phenotype | NoPhenotypeAvailable |
| Upregulated in class | na\_neg |
| GeneSet | DOROSZKO\_PERIVASCULAR\_INVASION |
| Enrichment Score (ES) | -0.75981075 |
| Normalized Enrichment Score (NES) | -2.4308994 |
| Nominal p-value | 0.0 |
| FDR q-value | 0.0 |
| FWER p-Value | 0.0 |
Table: GSEA Results Summary

  

Fig 1: Enrichment plot: DOROSZKO\_PERIVASCULAR\_INVASION      
 Profile of the Running ES Score & Positions of GeneSet Members on the Rank Ordered List

  

| PROBE | GENE SYMBOL | GENE\_TITLE | RANK IN GENE LIST | RANK METRIC SCORE | RUNNING ES | CORE ENRICHMENT || 1 | LMO4 |  |  | 305 | 0.230 | 0.0067 | No |
| 2 | CKB |  |  | 647 | 0.157 | -0.0029 | No |
| 3 | SNX1 |  |  | 1873 | 0.070 | -0.1213 | No |
| 4 | MAP2 |  |  | 2271 | 0.055 | -0.1543 | No |
| 5 | S100A10 |  |  | 2608 | 0.044 | -0.1824 | No |
| 6 | RHOB |  |  | 2690 | 0.042 | -0.1839 | No |
| 7 | GNG7 |  |  | 2967 | 0.035 | -0.2074 | No |
| 8 | GAS7 |  |  | 3074 | 0.032 | -0.2132 | No |
| 9 | SNX22 |  |  | 3545 | 0.023 | -0.2593 | No |
| 10 | ETV1 |  |  | 3908 | 0.017 | -0.2949 | No |
| 11 | RGS2 |  |  | 4038 | 0.015 | -0.3060 | No |
| 12 | S100A4 |  |  | 4483 | 0.008 | -0.3520 | No |
| 13 | NES |  |  | 6455 | -0.025 | -0.5573 | No |
| 14 | MEF2C |  |  | 6861 | -0.033 | -0.5948 | No |
| 15 | ANXA1 |  |  | 7265 | -0.044 | -0.6302 | No |
| 16 | PHLDA3 |  |  | 7590 | -0.053 | -0.6556 | No |
| 17 | F3 |  |  | 7662 | -0.055 | -0.6538 | No |
| 18 | DMD |  |  | 7987 | -0.067 | -0.6769 | No |
| 19 | PGM2L1 |  |  | 8768 | -0.118 | -0.7397 | Yes |
| 20 | BCHE |  |  | 8775 | -0.119 | -0.7201 | Yes |
| 21 | ANXA2 |  |  | 8891 | -0.134 | -0.7096 | Yes |
| 22 | MAP1B |  |  | 8945 | -0.141 | -0.6913 | Yes |
| 23 | PTN |  |  | 8989 | -0.147 | -0.6708 | Yes |
| 24 | S100A6 |  |  | 9097 | -0.166 | -0.6540 | Yes |
| 25 | DAG1 |  |  | 9192 | -0.187 | -0.6321 | Yes |
| 26 | PPFIBP1 |  |  | 9225 | -0.203 | -0.6010 | Yes |
| 27 | GSN |  |  | 9238 | -0.206 | -0.5673 | Yes |
| 28 | IGFBP2 |  |  | 9240 | -0.208 | -0.5321 | Yes |
| 29 | PCSK1N |  |  | 9321 | -0.247 | -0.4986 | Yes |
| 30 | PTPRZ1 |  |  | 9351 | -0.272 | -0.4553 | Yes |
| 31 | DKK1 |  |  | 9377 | -0.313 | -0.4046 | Yes |
| 32 | VCAN |  |  | 9396 | -0.364 | -0.3447 | Yes |
| 33 | TNC |  |  | 9403 | -0.383 | -0.2801 | Yes |
| 34 | COL6A1 |  |  | 9410 | -0.410 | -0.2110 | Yes |
| 35 | MEG3 |  |  | 9419 | -0.461 | -0.1335 | Yes |
| 36 | IGFBP5 |  |  | 9438 | -0.797 | 0.0002 | Yes |
Table: GSEA details [plain text format]

  

Fig 2: DOROSZKO\_PERIVASCULAR\_INVASION: Random ES distribution      
 Gene set null distribution of ES for **DOROSZKO\_PERIVASCULAR\_INVASION**
